# Analyzing circulating tumor cells and epithelial-mesenchymal transition status of papillary thyroid carcinoma patients following thyroidectomy: a prospective cohort study

**DOI:** 10.1097/JS9.0000000000001284

**Published:** 2024-03-04

**Authors:** Hyeong Won Yu, Eunju Park, Ja Kyung Lee, Woochul Kim, Ju Hyun Kong, Joseph Sunoo, Soon-Cheol Hong, June Young Choi

**Affiliations:** a Department of Surgery, Seoul National University Bundang Hospital; b CytoDx Inc., 331, Pangyo-ro, Bundan-gu, Seongnam, Gyeonggi-do; c Department of Surgery, Seoul National University College of Medicine, Jongno-gu, Seoul, Korea

**Keywords:** circulating tumor cell, CTC, liquid biopsy, papillary thyroid cancer, thyroidectomy

## Abstract

**Background:**

This study investigated the prevalence and subtype distribution of circulating tumor cells (CTCs) in patients with papillary thyroid cancer (PTC) before and after thyroidectomy to determine the potential of CTC count as a noninvasive marker of the efficacy of surgical treatment in PTC.

**Materials and methods:**

Between January 2021 and January 2022, 62 PTC patients who underwent thyroidectomy at Seoul National University Bundang Hospital were prospectively evaluated. Peripheral blood samples (7.5 ml) were collected from each patient for CTC analysis before surgery and at 2 weeks and 3 months after surgery. CTC count and the distribution of CTC subtypes, including epithelial, epithelial-mesenchymal, and mesenchymal phenotypes, were analyzed using the negative selection method and immunofluorescence staining. The relationship between CTC count and clinicopathological characteristics was analyzed before and after surgery.

**Results:**

Before surgery, CTCs were detected in 87% (54/62) of patients; the mean CTC count was 8.0 and the median was 5.0 in 7.5 ml of peripheral blood. The mesenchymal or epithelial-mesenchymal phenotypes were predominant. After thyroidectomy, the mean and median CTC count values decreased to 5.3 and 2.5, respectively, at 2 weeks and to 4.3 and 3.0, respectively, at 3 months. This postoperative reduction in CTCs was more pronounced in patients with lymphatic invasion, lymph node metastasis, or BRAF V600E mutation.

**Conclusion:**

CTCs were detected in patients with PTC with a predominance of cells undergoing epithelial-mesenchymal transition. The CTC count decreased postoperatively, suggesting that liquid biopsy with CTC detection could be a valuable noninvasive tool for monitoring the efficacy of surgery in PTC.

## Introduction

Highlights

- The presence of circulating tumor cells (CTC) before and after thyroidectomy using a negative enrichment technique and classifying epithelial, epithelial-mesenchymal, and mesenchymal CTC subtypes is unclear.
- We prospectively recruited 62 papillary thyroid cancer (PTC) patients who had operated on thyroidectomy for CTC detection.
- PTC patients had a significantly higher number of CTCs undergoing epithelial-mesenchymal transition, revealing the heterogeneity of the CTC population. Moreover, the number of CTCs decreased following cancer-removal surgery.

Papillary thyroid cancer (PTC) is the most common thyroid malignancy, accounting for ~80% of all thyroid cancer cases^1^. Despite its generally favorable prognosis, PTC can pose clinical challenges, particularly in cases of aggressive behavior or recurrence^2^. The primary treatment modality is surgery, which ranges from partial (such as lobectomy) to complete thyroidectomy (total thyroidectomy)^3,4^, and effective postoperative surveillance is essential to detect recurrence or metastasis. Traditionally, this monitoring has relied on noninvasive methods such as serum thyroglobulin tests and neck ultrasonography^5–7^. Thyroglobulin is a biomarker that is usually found at low or undetectable levels following total thyroidectomy^8^. However, in procedures such as isthmectomy, which are associated with residual thyroid tissues, thyroglobulin levels may remain detectable because of the presence of benign thyroid tissue, potentially leading to unclear results^9,10^. Additionally, although neck ultrasonography can detect structural recurrences, very small or early microscopic metastases may remain undetected^11^.

Liquid biopsy, mostly the detection of circulating tumor cells (CTCs), provides a promising alternative for the sensitive detection of residual or recurrent cancer^12,13^. CTCs, which are shed from primary tumors into the bloodstream, are indicators of ongoing pathological processes and could provide insight into the metastatic potential of tumors^14^. Their detection offers a more sensitive approach to identifying residual or recurrent cancer than that of conventional biomarkers. This method can complement existing strategies by providing detailed, patient-specific information on the presence and characteristics of tumor cells postsurgery. Such data are crucial for guiding subsequent therapeutic decisions and tailoring follow-up care to individual patient needs, which should improve the management and prognosis of PTC. However, the role and dynamics of CTCs in the post-thyroidectomy monitoring of PTC patients remain under-investigated, highlighting the need for further research in this area.

In this prospective cohort study, we examined the effectiveness of CTC detection for monitoring PTC patients undergoing thyroidectomy by comparing CTC count before and after surgery. We hypothesized that monitoring CTCs might provide valuable information on disease progression and the potential for metastasis, thereby contributing to postoperative surveillance.

## Materials and methods

### Study design

This study prospectively enrolled a cohort of patients who underwent thyroidectomy for PTC between January 2021 and January 2022 at Seoul National University Bundang Hospital (SNUBH). All operations were performed by two surgeons (JY Choi and HW Yu). Patients were excluded if they underwent a simultaneous operation in addition to thyroidectomy. Finally, a total of 62 patients were included in the analysis. Peripheral blood samples were collected to analyze CTCs before thyroidectomy and at 2 weeks and 3 months after thyroidectomy. CTC counts and the distribution of CTC subtypes before and after surgery were analyzed using the negative selection method and immunofluorescence staining. The relationship between CTC count and clinicopathological characteristics was statistically analyzed before and after surgery.

This study was approved by the institutional review board of SNUBH (IRB No.: B-1612/374-303) and performed in compliance with its guidelines. The trial was registered on the Clinical Research Information Service (KCT0008179, Date of registration: 10/02/2023, www.cris.nih.go.kr) approved by the WHO International Clinical Trials Registry Platform. The work was reported following the strengthening the reporting of cohort, cross-sectional, and case–control studies in surgery (STROCSS) criteria^15^.

The aim of the study was to examine the potential of CTC count as a noninvasive marker for monitoring the effectiveness of surgical treatment in patients with PTC.

### Data collection

Demographic information, surgical data, and the information contained in the pathology reports were collected from patients’ medical records. BMI was calculated using height and weight measurements. BRAF gene mutation status and CTC information were collected and recorded.

### Enumeration of CTCs

CTC enrichment and characterization were performed using the CytoDx CTC detection system (CytoDx Inc., Korea). Briefly, 7.5 ml of peripheral blood were collected from each patient by venipuncture before surgery and at 2 weeks and 3 months after surgery. Peripheral blood mononuclear cells (PBMCs) and CTCs were isolated by Ficoll-Paque (Cytiva, USA) density gradient centrifugation. Before immunocytochemical staining, PBMCs were removed using antibodies against CD16 (BioLegend, USA), CD19 (Thermo-Fisher Scientific, USA), CD45 (Thermo-Fisher Scientific), CD163 (Thermo-Fisher Scientific), and CD235a (Thermo-Fisher Scientific). For immunofluorescence staining, PMBC-depleted cells were plated on microscope slides (Paul Marienfeld, Germany). Cells were stained with fluorescent dye-labeled primary antibodies [Alexa Fluor 647-conjugated anti-EpCAM antibody (Santa Cruz, USA), Alexa Fluor 647-conjugated anti-pan-CK antibody (Novus Biologicals, USA), and DyLight550-conjugated anti-vimentin antibody (Novus Biologicals)] and biotin-conjugated primary antibodies against CD16 (BioLegend), CD19 (Thermo-Fisher Scientific), CD45 (Thermo-Fisher Scientific), and CD163 (Thermo-Fisher Scientific). After several washes, cells were incubated with the secondary antibodies streptavidin Alexa Fluor 488 (Thermo-Fisher Scientific) and Hoechst 33342 (Thermo-Fisher Scientific) for DNA staining. Fluorescence signals were visualized using the Olympus SLIDEVIEW VS200 Research Slide Scanner (EVIDENT, Japan).

### Identification of CTCs in PTC patients

**Figure 1:**
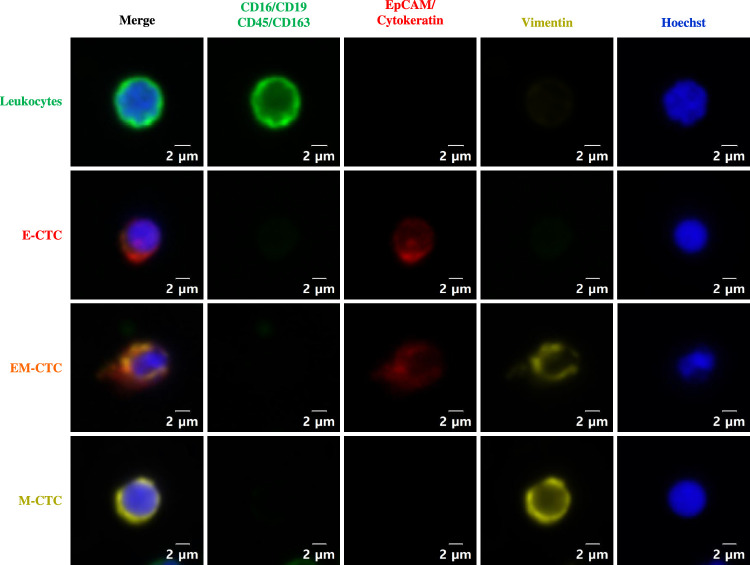
Identification of circulating tumor cells in papillary thyroid cancer patients. Leukocytes are positive for CD16, CD19, CD45, and/or CD163; E-CTC is positive for EpCAM and/or cytokeratin but is negative for vimentin; M-CTC is positive for vimentin but is negative for EpCAM and cytokeratin; EM-CTC is positive for both EpCAM/cytokeratin and vimentin. All live cells were positive for Hoechst staining. CTC, circulating tumor cell; E-CTC, epithelial CTC; EM-CTC, epithelial-mesenchymal CTC; M-CTC, mesenchymal CTC.

Cells were defined as CTCs if they met the following criteria: round to oval morphology, a visible nucleus by Hoechst 33342 staining, positive staining for either epithelial cell markers (EpCAM and cytokeratin) or a mesenchymal cell marker (vimentin), and negative staining for leukocyte cell markers (CD16, CD19, CD45, and CD163). As shown in Figure 1, CTCs were negative for all leukocyte markers and were divided into three subtypes according to EpCAM/cytokeratin and vimentin expression as follows: epithelial CTCs (E-CTCs) were positive for EpCAM/cytokeratin and negative for vimentin, mesenchymal CTCs (M-CTCs) showed the opposite pattern, and epithelial-mesenchymal CTCs (EM-CTCs) were positive for both EpCAM/cytokeratin and vimentin.

### Statistical analysis

Data were analyzed using GraphPad Prism 9.0.2 (GraphPad Software Inc.). Comparisons between two groups of continuous data were performed by the Mann–Whitney *U* test, and the *χ*^2^ test was used for categorical data. Multiple groups were compared using the Kruskal–Wallis test followed by Dunn’s multiple comparisons test. All statistical analyses were two-sided, and *P*<0.05 was considered significant.

## Results

### Clinicopathological characteristics of PTC patients

The study included 62 patients with PTC. The clinical and pathological characteristics of the patients are summarized in Tables 1 and 2. Of the 62 patients, 26 (41.9%) were male. The mean age of the prospective cohort at the time of sampling was 45.55±12.10 years. The mean BMI was 24.90±3.46. Of the 62 surgical patients, 21 (33.9%) underwent conventional open thyroidectomy, 6 (9.7%) underwent isthmectomy, and 35 (56.5%) underwent lobectomy. Central lymph node dissection was performed in 54 (87.1%) and lateral lymph node dissection was performed in 10 (16.1%) patients. The average size of the tumor mass was 1.70±1.32 cm (Table 2). Conventional, follicular, tall cell, and oncocystic subtypes were detected in 34 (54.8%), 14 (22.6%), 12 (19.4%), and 2 (3.2%) patients, respectively. Blood vessel invasion, lymphatic invasion, and lymph node metastasis were observed in 3 (4.8%), 43 (69.4%), and 32 (51.6%) patients, respectively, and 43 (69.3%) patients had BRAF V600E mutation.

**Table 1:** Baseline clinical characteristics of 62 papillary thyroid cancer patients.

Clinical characteristics	All patients (*N*=62) *N* (%)
Sex	
Male	26 (41.9)
Female	36 (58.1)
Age (years; mean ±SD)	45.55±12.10
BMI (kg/m^2^; mean ±SD)	24.90±3.46
Surgical method	
Open	21 (33.9)
Robot	41 (66.1)
Extent of surgery	
Isthmectomy	6 (9.7)
Lobectomy	35 (56.5)
Total thyroidectomy	21 (33.9)
Extent of cLND	
Ipsilateral	34 (54.8)
Bilateral	20 (32.3)
Not done	8 (12.9)
Lateral LND	
Yes	10 (16.1)
No	52 (83.9)

cLND, central lymph node dissection; LND, lymph node dissection.

**Table 2:** Baseline pathological characteristics of papillary thyroid cancer patients.

Pathological characteristics	All patients (*N*=62) *N* (%)
Main mass tumor size (cm; mean±SD)	1.70±1.32
PTC variant	
Conventional	34 (54.8)
Follicular	14 (22.6)
Tall cell	12 (19.4)
Oncocystic	2 (3.2)
Multicentricity	
Present	15 (24.2)
Absent	47 (75.8)
Extrathyroidal extension	
Gross	2 (3.2)
Minimal	3 (4.8)
Microscopic	30 (48.4)
None	27 (43.5)
Blood vessel invasion	
Present	3 (4.8)
Absent	59 (95.2)
Lymphatic invasion	
Present	43 (69.4)
Absent	19 (30.6)
Lymph node metastasis	
Present	32 (51.6)
Absent	16 (25.8)
Unknown	14 (22.6)
BRAF mutation	
Positive	43 (69.3)
Negative	14 (22.6)
Not done	5 (8.1)

PTC, papillary thyroid cancer.

### The CTC count is increased in patients with PTC

**Figure 2:**
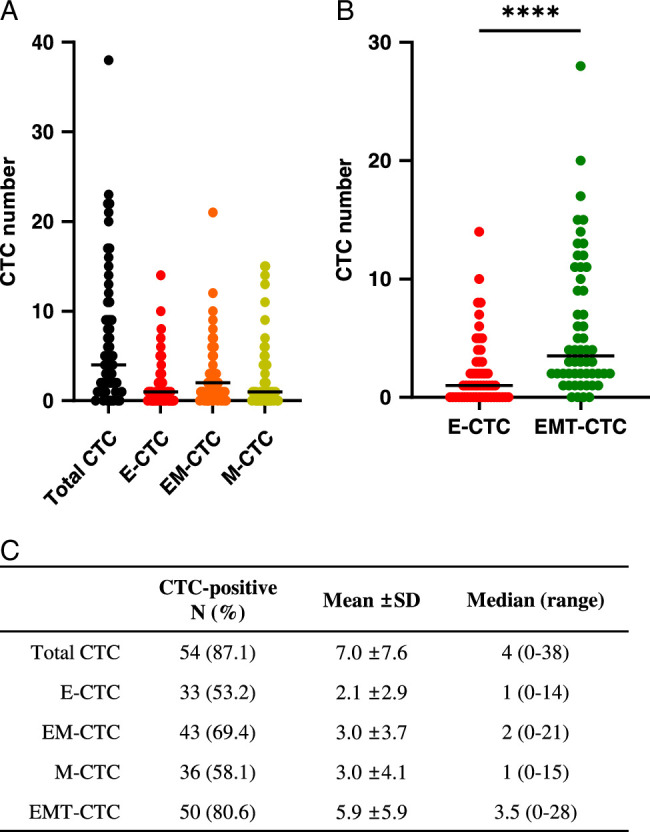
Patients with papillary thyroid cancer show a significantly higher number of CTCs undergoing epithelial-mesenchymal transition. (A) Distribution of total CTCs, E-CTCs, EM-CTCs, and M-CTCs in papillary thyroid cancer patients. (B) A significant difference in distribution between E-CTCs and EMT-CTCs in papillary thyroid cancer patients. (C) Mean and median numbers of CTC subtypes in papillary thyroid cancer patients. CTC, circulating tumor cell; E-CTC, epithelial CTC; EM-CTC, epithelial-mesenchymal CTC; M-CTC, mesenchymal CTC; EMT-CTC, a combined group of EM-CTC and M-CTC; ****P-value<0.0001.

To determine whether CTCs are present in PTC patients, 7.5 ml of peripheral blood were collected from each of the 62 PTC patients and analyzed using the CytoDx CTC enumeration system. This system allows the identification of three CTC subtypes, E-CTCs, EM-CTCs, and M-CTCs, by detecting the expression of epithelial and mesenchymal cell markers with fluorescent dyes (Fig. 1). CTCs were detected in 87% (54/62) of PTC patients with a mean number of 7.0 and a median of 5.0 in 7.5 ml of peripheral blood (Fig. 2A and C). E-CTCs, EM-CTCs, and M-CTCs were detected in 53% (33/62), 69% (43/62), and 58% (36/62) of patients, respectively, and EM-CTCs and M-CTCs were more abundant than E-CTCs. The combined population of EM-CTCs and M-CTCs (EMT-CTCs) was significantly higher than that of E-CTCs, with a threefold higher number of cells (5.9 vs. 2.1, *P*<0.0001). Of 54 CTC-positive patients, 50 had EMT-CTCs and 4 were E-CTC-only patients, indicating that in the majority (93%) of PTC patients, CTCs were undergoing epithelial-mesenchymal transition (EMT) (Fig. 2B–C).

### Correlation of CTCs with baseline clinicopathological characteristics of PTC patients

**Figure 3:**
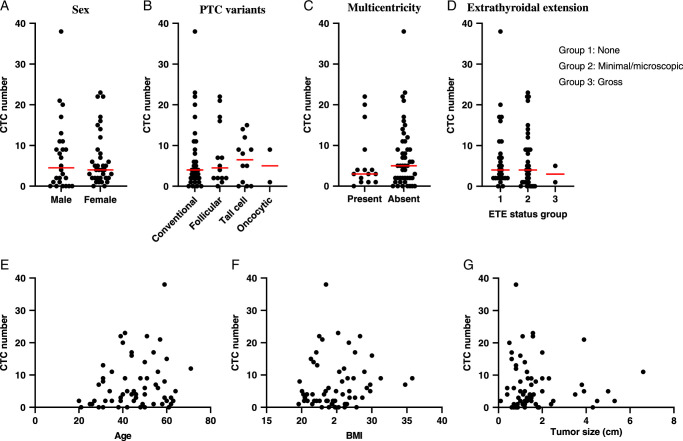
Association of total CTC number and clinicopathological features of PTC patients: (A) sex, (B) PTC variants, (C) multicentricity, (D) extrathyroidal extension, (E) age, (F) BMI, and (G) main mass tumor size. A red line indicates a median number of total CTC. ETE, extrathyroidal extension; PTC, papillary thyroid cancer.

Next, we analyzed the correlation between CTC positivity and the baseline clinicopathological features of PTCs (Table 3). We found that CTC positivity was not correlated with the surgical method, the extent of surgery, lymph node dissection, type of PTC variants, multicentricity, degree of extrathyroidal extension, blood vessel invasion, lymphatic invasion, lymph node metastasis, and BRAF mutation (*P*>0.05). Similar results were obtained in the analysis of the association between CTC number and the clinicopathological features of PTCs such as sex, type of PTC variant, multicentricity, degree of extrathyroidal extension, and patient age and BMI (Fig. 3), which were not correlated with the total CTC count. Tumor size was not correlated with the total CTC count. Although the total CTC number was not associated with sex, the CTC positivity rate was significantly higher in women than in men (*P*<0.05) (Table 3).

**Table 3:** Comparison of baseline clinicopathological characteristics between CTC-positive and CTC-negative PTC patients.

Clinicopathological characteristics	CTC-positive (*N*=54; 87.1%) *N* (%)	CTC-negative (*N*=8; 12.9%) *N* (%)	*P* ^*^
Sex			0.042
Male	20 (37.0)	6 (75.0)	
Female	34 (63.0)	2 (25.0)	
Surgical method			0.570
Open	19 (35.2)	2 (25.0)	
Robot	35 (64.8)	6 (75.0)	
Extent of surgery			0.611
Isthmectomy	6 (11.1)	0 (0.0)	
Lobectomy	30 (55.6)	5 (62.5)	
Total thyroidectomy	18 (33.3)	3 (37.5)	
Extent of cLND			0.733
Ipsilateral	30 (55.5)	4 (50.0)	
Bilateral	17 (31.5)	3 (37.5)	
Not done	7 (13.0)	1 (12.5)	
Lateral LND			0.465
Yes	8 (14.8)	2 (25.0)	
No	46 (85.2)	6 (75.0)	
PTC variant			0.510
Conventional	30 (55.5)	4 (50)	
Follicular	13 (24.1)	1 (12.5)	
Tall cell	9 (16.7)	3 (37.5)	
Oncocystic	2 (3.7)	0 (0.0)	
Multicentricity			0.408
Present	14 (25.9)	1 (12.5)	
Absent	40 (74.1)	7 (87.5)	
Extrathyroidal extension			0.422
Gross	2 (3.7)	0 (0.0)	
Minimal	3 (5.6)	0 (0.0)	
Microscopic	24 (44.4)	6 (75.0)	
None	25 (46.3)	2 (25.0)	
Blood vessel invasion			0.494
Present	3 (5.6)	0 (0.0)	
Absent	51 (94.4)	8 (100.0)	
Lymphatic invasion			0.233
Present	36 (66.7)	7 (87.5)	
Absent	18 (33.3)	1 (12.5)	
Lymph node metastasis			0.247
Present	26 (48.1)	6 (75.0)	
Absent	15 (27.8)	1 (12.5)	
Unknown	13 (24.1)	1 (12.5)	
BRAF mutation			0.359
Positive	38 (70.4)	5 (62.5)	
Negative	11 (20.4)	3 (37.5)	
Not done	5 (9.2)	0 (0.0)	

cLND, central lymph node dissection; CTC, circulating tumor cell; LND, lymph node dissection; PTC, papillary thyroid cancer.

* chi-square test.

### Total CTC count decreases after surgery

**Figure 4:**
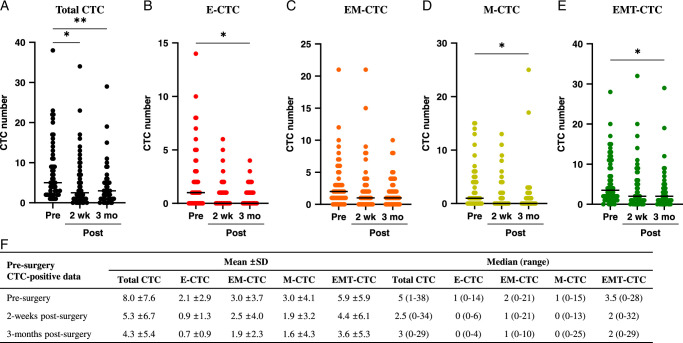
CTC count significantly decreases after surgery in presurgery CTC-positive papillary thyroid cancer patients. Distribution of (A) total CTC, (B) E-CTC, (C) EM-CTC, (D) M-CTC, and EMT-CTC at presurgery, 2 weeks post-surgery, and 3 months post-surgery. (F) Table summarizing mean and median numbers of CTC subtypes shown in (A)-(E). CTC, circulating tumor cell; E-CTC, epithelial CTC; EM-CTC, epithelial-mesenchymal CTC; M-CTC, mesenchymal CTC; EMT-CTC, a combined group of EM-CTC and M-CTC; *P-value <0.05, **P-value <0.01.

To determine the effect of surgical tumor removal on the number of CTCs, we measured the CTC count before surgery and at 2 weeks and 3 months after surgery in the 54 PTC patients who were CTC-positive before surgery. As shown in Figure 4, the CTC count decreased significantly after surgery, showing a reduction in the mean count from 8.0 before surgery to 5.3 and 4.3 at 2 weeks and 3 months postsurgery, respectively. Similarly, the median count decreased from 5.0 before surgery to 2.5 and 3.0 at 2 weeks and 3 months postsurgery, respectively. All CTC subtypes showed reduced numbers after surgery. When we combined EM-CTCs and M-CTCs as EMT-CTCs, the number significantly decreased 2 weeks and 3 months after surgery (Fig. 4E–F).

**Figure 5:**
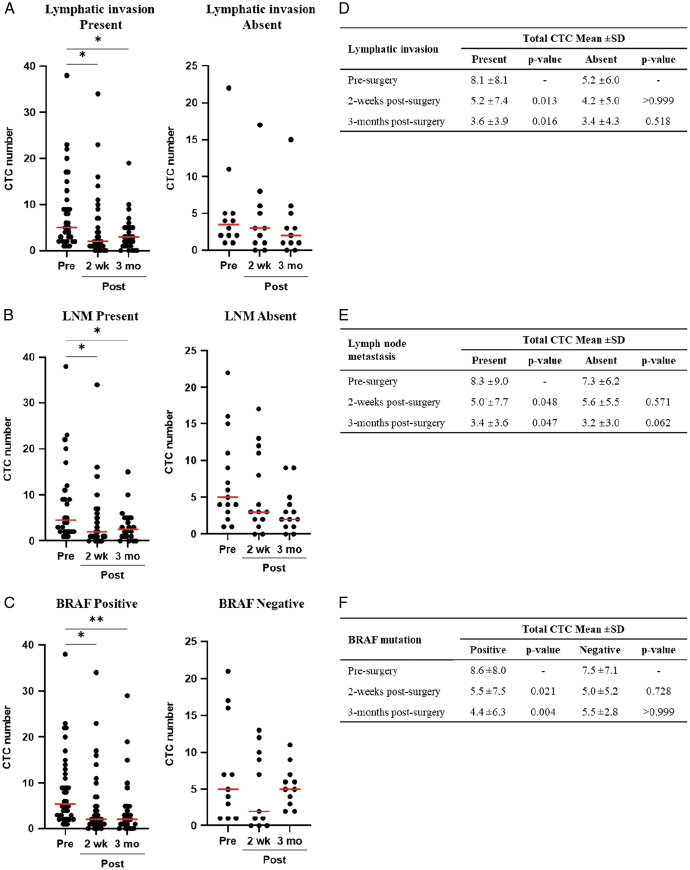
PTC patients with lymphatic invasion, lymph node metastasis, or BRAF V600E mutation show significantly decreased CTC count after surgery. Distribution of total CTCs at presurgery, two weeks postsurgery, and 3 months postsurgery according to (A) lymphatic invasion, (B) lymph node metastasis, and (C) BRAF V600E mutation. (D–F) Mean numbers of total CTCs and comparison of according to (A) lymphatic invasion, (B) lymph node metastasis, and (C) BRAF V600E mutation. CTC, circulating tumor cell; LNM, lymph node metastasis; *P-value <0.05; **P-value <0.01.

Next, we identified the baseline clinical and pathological characteristics affecting the changes in CTC count after surgery. The results showed that the CTC count decreased significantly in PTC patients with lymphatic invasion compared with that in patients without lymphatic invasion (*P*<0.05, Fig. 5A and D). Similarly, the decrease in CTC number was greater in patients with lymph node metastasis (*P*<0.05, Fig. 5B and E) and BRAF V600E mutation (*P*<0.01, Fig. 5C and F) than in those without.

## Discussion

The results of this study indicate that enumeration of CTCs could be valuable for the therapeutic monitoring of PTC patients. We observed a high prevalence of CTCs in 87% of PTC patients before surgery, which is consistent with findings in other cancers such as breast^16^ and lung cancer^17^. This suggests the potential of CTCs as biomarkers in PTC, although additional research is required to reach a definitive conclusion. We also noted a significant reduction in CTCs after surgery, especially in patients with aggressive PTC characteristics such as lymphatic invasion and BRAF V600E mutation (Figs 4, 5). This highlights the potential of CTC enumeration for evaluating the efficacy of surgical intervention.

We used a negative selection approach to CTC detection to analyze various CTC subtypes in PTC. This method overcomes the limitations of techniques based on epithelial markers (EpCAM) and size^18,19^, thus allowing the identification of CTCs of any size, including those undergoing EMT^20^. EMT is a crucial factor in cancer metastasis and progression^21^. We found a high prevalence of EMT-CTCs at baseline before surgery, when they were detected in 93% of PTC patients, with a notable reduction following surgery. This finding supports the effect of surgical intervention on the EMT process in PTC and contributes to our understanding of tumor heterogeneity.

This study has several limitations. The relatively small sample size and the single-institution analysis may affect the generalizability of the findings. A more diverse cohort across multiple centers would provide more robust conclusions. In addition, the 3-month postsurgery follow-up may not fully capture the long-term CTC trend in PTC. Future longitudinal studies should aim to track the dynamic changes over extended periods of time to better understand their correlation with patient outcomes such as recurrence and survival rates. The lack of a control group, including individuals with benign thyroid conditions or healthy subjects, also limits the comparative analysis. To address these shortcomings, we are currently conducting clinical trials with a diverse pool of participants, including those with benign tumors and various types of thyroid cancer. These efforts aim to deepen our understanding of CTCs in PTC to provide information for effective clinical interventions.

In conclusion, this study highlights the value of CTC enumeration for monitoring surgical outcomes in PTC. The findings suggest that additional research is necessary to explore and validate the clinical application of CTC enumeration for the comprehensive management of PTC.

## Ethical approval

This study was approved by the institutional review board of SNUBH (IRB No.: B-1612/374-303) and performed in compliance with its guidelines. The trial was registered on the Clinical Research Information Service (KCT0008179, Date of registration: 10/02/2023, www.cris.nih.go.kr), which the WHO International Clinical Trials Registry Platform approves.

## Sources of funding

This work was supported by grant 13-2018-016 and 06-2020-354 from the Seoul National University Bundang Hospital Research Fund, Republic of Korea.

## Author contribution

H.W.Y.: conceptualization, writing–original draft, and writing–review and editing; E.P.: writing–original draft and data analysis; J.K.L. and W.K.: data curation and software; J.H.K.: CTC enumeration; S.-C.H.: conceptualization and writing–review and editing; J.S.: conceptualization and writing–review; J.Y.C.: conceptualization, supervision, writing–review and editing, and project administration.

## Conflicts of interest disclosure

The authors declare no competing interests.

## Research registration unique identifying number (UIN)

The trial was registered on the Clinical Research Information Service (KCT0008179, Date of registration: 10/02/2023, www.cris.nih.go.kr), which the WHO International Clinical Trials Registry Platform approves.

## Guarantor

June Young Choi.

## Data availability statement

The data that support these findings are available upon request from the corresponding author.

## Provenance and peer review

Not commissioned, externally peer-reviewed.

## Additional information

Correspondence and requests for materials should be addressed to J.Y.C.
